# High-resolution topography reveals morphological changes of Stromboli volcano following the July 2024 eruption

**DOI:** 10.1038/s41597-024-04098-y

**Published:** 2024-11-12

**Authors:** Riccardo Civico, Tullio Ricci, Alessandro Cecili, Piergiorgio Scarlato

**Affiliations:** 1 Istituto Nazionale di Geofisica e Vulcanologia, Rome, Italy; 2 Università degli studi Roma Tre, Dipartimento di Scienze, Rome, Italy

**Keywords:** Volcanology, Natural hazards

## Abstract

The July 2024 eruption of Stromboli volcano has been characterised by the manifestation, at variable intensity, of the entire repertoire of volcanic events that Stromboli volcano is capable of, and is by far the one that has most changed the morphology of the crater terrace and of the Sciara del Fuoco slope in the last decades. We present the results of an Unoccupied Aircraft System (UAS) survey performed on 14 July 2024 and consisting of 4,988 visible and thermal photographs coupled with Structure-from-Motion photogrammetry that allowed us to produce a high-resolution (0.2 m/pixel) Digital Surface Model (DSM). We documented the profound morphological changes of the Stromboli volcano resulting from the 4–11 July 2024 eruption and obtained elevation and volume change estimates by differencing our survey and a UAS-derived pre-event surface (24 May 2024).

## Background & Summary

Dynamic processes such as eruptive activity, erosion and mass wasting events continuously shape the morphology of active volcanoes. Consequently, updated high-resolution digital elevation models (DEMs) are a fundamental tool for many applications, with mapping and hazard assessment, as well as initialisation of numerical models, among the most important ones.

Unoccupied Aircraft Systems (UAS) have emerged over the past decade as an increasingly powerful and reliable means to provide detailed and invaluable aerial observation promptly. Applying Structure-from-Motion (SfM)^1,2^ to UAS-derived imagery datasets permits the production of high-resolution DEMs^3–9^, minimising risk to personnel and crewed overflight costs.

**Fig. 1:**
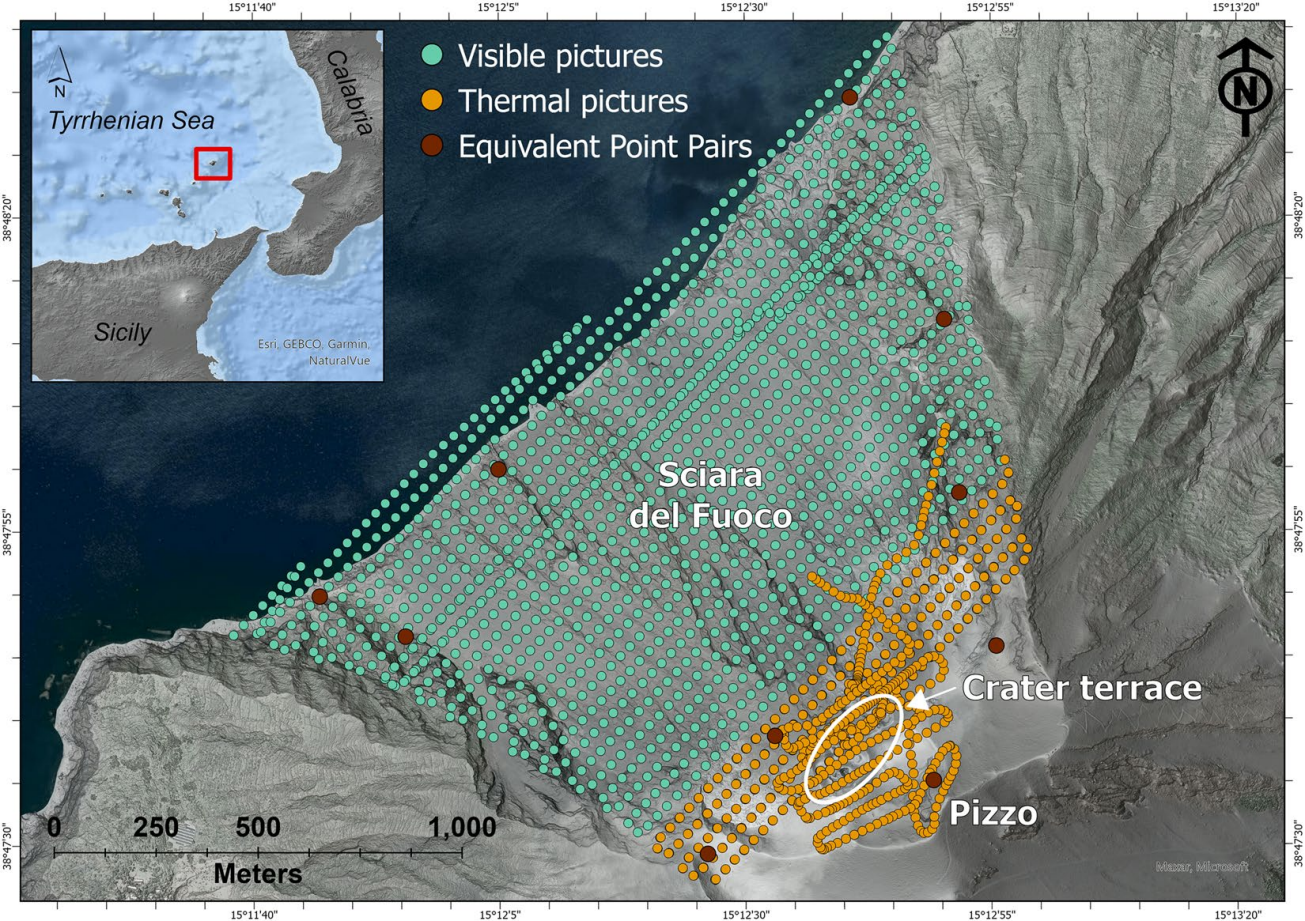
Map identifying the location of each image acquired during the survey. Green dots represent visible pictures, orange dots are thermal images. Brown dots are the equivalent point pairs used for point cloud alignment. The inset at the top left of the figure shows the location of Stromboli Island (red square) within the southern Tyrrhenian Sea.

Stromboli volcano, located in the Southern Tyrrhenian Sea (Italy) off the northern coast of Sicily (Fig. 1), is one of the world’s most active, most studied and well-monitored volcanoes. Its activity is characterised by frequent impulsive low-to-moderate energy explosions from the summit craters, named Strombolian activity. This ordinary eruptive activity is occasionally interrupted by higher-energy explosions (Major Explosions and Paroxysmal Eruptions^10^; and references therein). Volcanic hazards at Stromboli^10^ include ballistic and ash fall-out, lava flows, pyroclastic density currents, rockfalls and landslides, gas and aerosol emissions (including vog and laze), acid rains, and volcano-related wildfires and tsunamis.

**Fig. 2:**
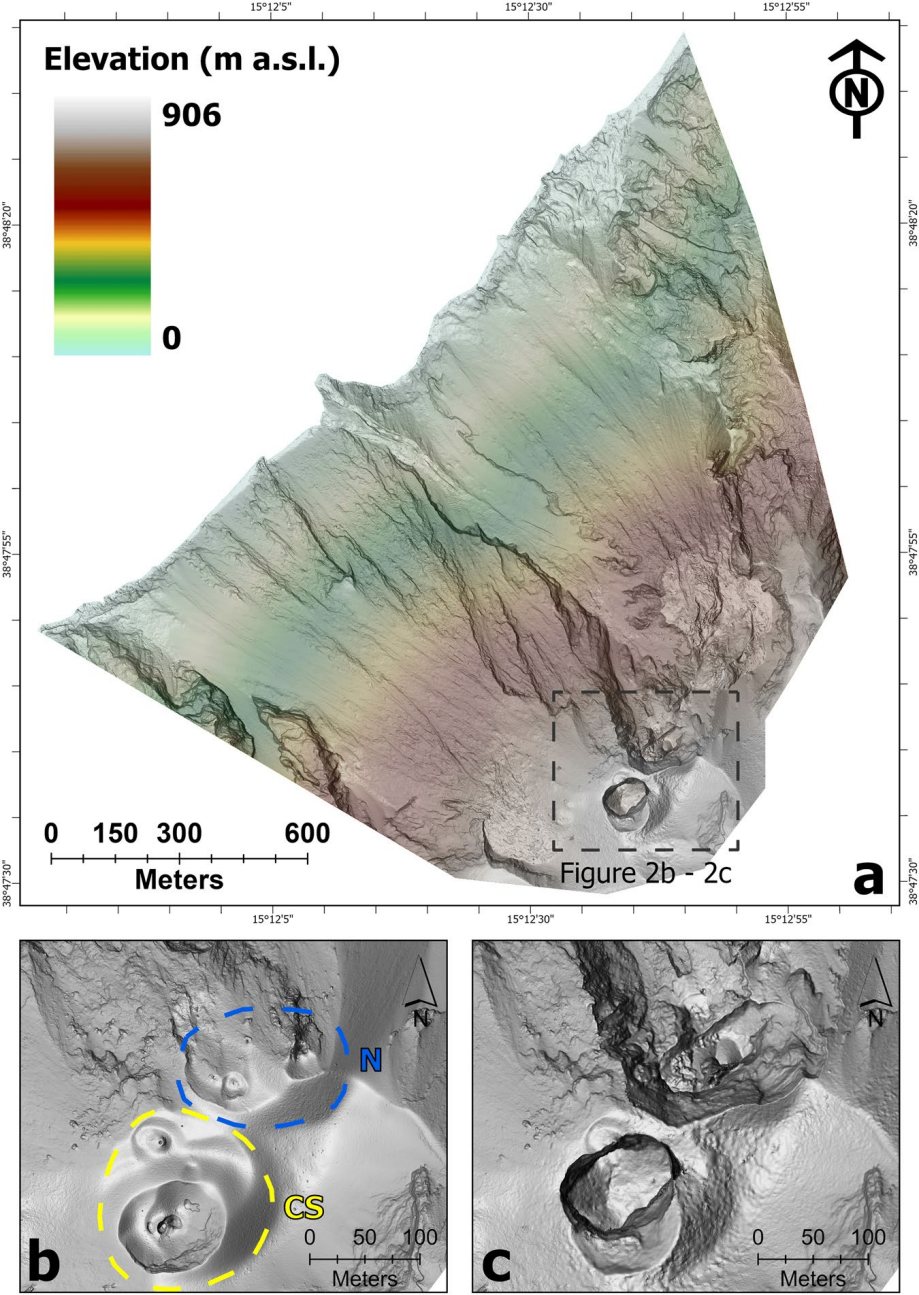
Digital Surface Model (DSM) of the Sciara del Fuoco and crater terrace at Stromboli volcano following the July 2024 eruption. (**a**) Multidirectional hillshade of the 14 July DSM^17^. (**b**) Detailed view of the crater terrace (N and CS are North and Central South crater sectors, respectively) on 24 May 2024^18^ and (**c**) on 14 July 2024^17^.

In recent years, detailed UAS-based photogrammetric surveys have been carried out at Stromboli volcano at the scale of the crater terrace^11–13^ (conventionally divided in North and Central South crater sectors, N and CS in Fig. 2b) and, more recently, the Sciara del Fuoco has also been included^14–16^.

The 4–11 July 2024 eruption of Stromboli volcano has been characterised by the manifestation, at variable intensity, of the entire repertoire of volcanic events that Stromboli volcano is capable of, apart from the mild ordinary Strombolian explosive activity which stopped at the onset of the eruption.

The eruptive events began at 14:10 (UTC) on 4 July and, until 5 July included lava flows, mass wasting events, pyroclastic density currents (https://youtu.be/6wCw4VWDyjs?feature=shared), the opening of new vents, and the generation of small tsunami waves. The lava flows, forming a lava delta on the shoreline, continued until 11 July when a paroxysmal eruption occurred at 12:08 (UTC), interrupting the effusive activity and ending the eruption. The eruption was preceded, between the afternoon of 3 July and the morning of the 4th, by Strombolian and spattering activity, small-volume collapse events affecting the N crater area, a weakly fed lava overflow and a major explosion.

The July 2024 eruption of Stromboli volcano is by far the one that has most changed the morphology of the crater terrace in the last decades. The most significant morphological changes occurred on the afternoon of the 4 July, whereas no noteworthy morphological modifications appear to have occurred following the 11 July paroxysmal eruption. The maximum differences in altitude, recorded in the N sector correspond to a variation of over 100 m compared to the previous survey (elevation of lower N crater vent approximately 700 m a.s.l. vs. previous elevation of approximately 805 m a.s.l.).

Here, we present the results of a UAS survey carried out on 14 July 2024 using a DJI Mavic 3T RTK (Real-Time Kinematic). The infrared thermal and visible aerial images were georeferenced using an onboard RTK receiver capable of cm-level positioning accuracy. The dataset was then processed using Structure-from-Motion (SfM) photogrammetry to generate a high-resolution (0.2 m/pixel) Digital Surface Model (DSM), covering the crater terrace and the entire Sciara del Fuoco (Fig. 1) and revealing the new morphology of the Stromboli volcano resulting from the 4–11 July 2024 eruption.

Topographic change detection was obtained by differencing our 14 July^17^ survey and a pre-eruption (24 May 2024) 0.2 m/pixel UAS-derived DSM^18^ and highlighted the profound morphological changes of the Stromboli volcano following the 4–11 July 2024 eruption.

Summary of the main findings:

- We estimated the total subaerial volume loss to be 6.03 ± 0.15 Mm^3^. Of this volume, the canyon accounted for 2.74 ± 0.07 Mm^3^.
- We estimated the total subaerial volume gain (lava flow, PDC, fallout and landslide deposits) to be 0.93 ± 0.14 Mm^3^. This volume is a minimum estimate, as the DSM does not take into account the submerged volume.

## Methods

### UAS survey and DSM generation

We conducted a UAS survey campaign (Fig. 1 and Table 1) on 14 July 2024, acquiring simultaneously 4,988 aerial thermal and visible photographs using a DJI Mavic 3T RTK equipped with infrared thermal and visible cameras. A total of 8 multi-battery missions were conducted for the survey from the same take-off point, for around 160 minutes and a cumulative flight path of over 60 km (Fig. 1). All flights except one were nadir image data collection flights, conducted at an altitude of 250 metres above ground level (a.g.l.) using predefined flight missions and the terrain follow feature. Flight planning was designed with 80% forward and side overlap at ground level. A persistent vapour/gas and ash plume being emitted by the crater area during our flights strongly limited the visibility and safety of the drone, preventing us from performing predefined flight missions in the summit area. To overcome this drawback, we conducted a manual flight over the crater terrace (altitude variable between 100 and 250 metres a.g.l.) collecting both nadir and oblique images (Fig. 1).

**Table 1:** Details of the photogrammetric survey data and elaboration. *Only for the predefined flight missions.

	Visible images survey	Infrared Thermal images survey
Flying altitude (m a.g.l.)	250	100–250
Forward overlap (%)	80	80*
Side overlap (%)	80	80*
Acquired images	2,494	2,494
Selected images	1,582	570
Aligned images	1,580	570
Sensor resolution	4000 × 3000	640 × 512
Ground resolution (cm/pixel)	9.1 cm/pixel	31.4 cm/pixel
Photo alignment accuracy	High	Ultra High
Key point limit	40,000	40,000
Tie point limit	10,000	10,000
Depth maps generation quality	High	Ultra High
Depth maps filtering mode	Aggressive	Aggressive
Tie points	1,680,432	294,422
Point cloud	121,445,408	9,090,135

Data on camera position were collected using GNSS-RTK information embedded in the image metadata, with differential corrections sent in real-time by the “Sicili@net” service (www.ct.ingv.it/index.php/risorse-e-servizi/sicil-net, accessed on 12 August 2024) provided by the Istituto Nazionale di Geofisica e Vulcanologia (INGV). Camera positions have an accuracy of 1–2 cm in horizontal coordinates and 2–7 cm in elevation.

The poor visibility on the summit area (crater terrace and Pizzo), prevented us from using visible images to reconstruct the crater terrace; to overcome this drawback and maximise the reconstruction of the entire morphology of the Sciara del Fuoco and the crater terrace we decided to blend thermal and visible surveys. Following image collection, we culled the photoset, removing dark and/or blurry photos. We then processed separately 1,582 visible and 570 infrared thermal aerial georeferenced images using the Agisoft Metashape Pro® software package (version 2.1.2) based on the Structure-from-Motion and multi-view stereo photogrammetry algorithm (SfM–MVS)^1^. The workflow of our photogrammetric analysis is similar to that of Civico *et al*.^4^ and included the following: (1) image masking for areas with strong degassing and/or unnecessary background; (2) camera triangulation with image position and orientation and generation of sparse point cloud; (3) filtering of the sparse point cloud to remove points with bad geometry, large reprojection errors, and large reconstruction uncertainty; (4) generation of the dense point cloud; (5) cleaning of the dense point cloud by removing low confidence points; 6) merging of the two point clouds resulting from visible and thermal images, respectively; 7) point cloud alignment to an airborne lidar reference (see “Technical Validation) section.

Following processing and alignment of the dataset, we used our point cloud to generate a 0.2 m/pixel DSM (Fig. 2) using the DEM tool in Agisoft Metashape Pro® (inverse-distance weighting method).

### Elevation change detection

**Fig. 3:**
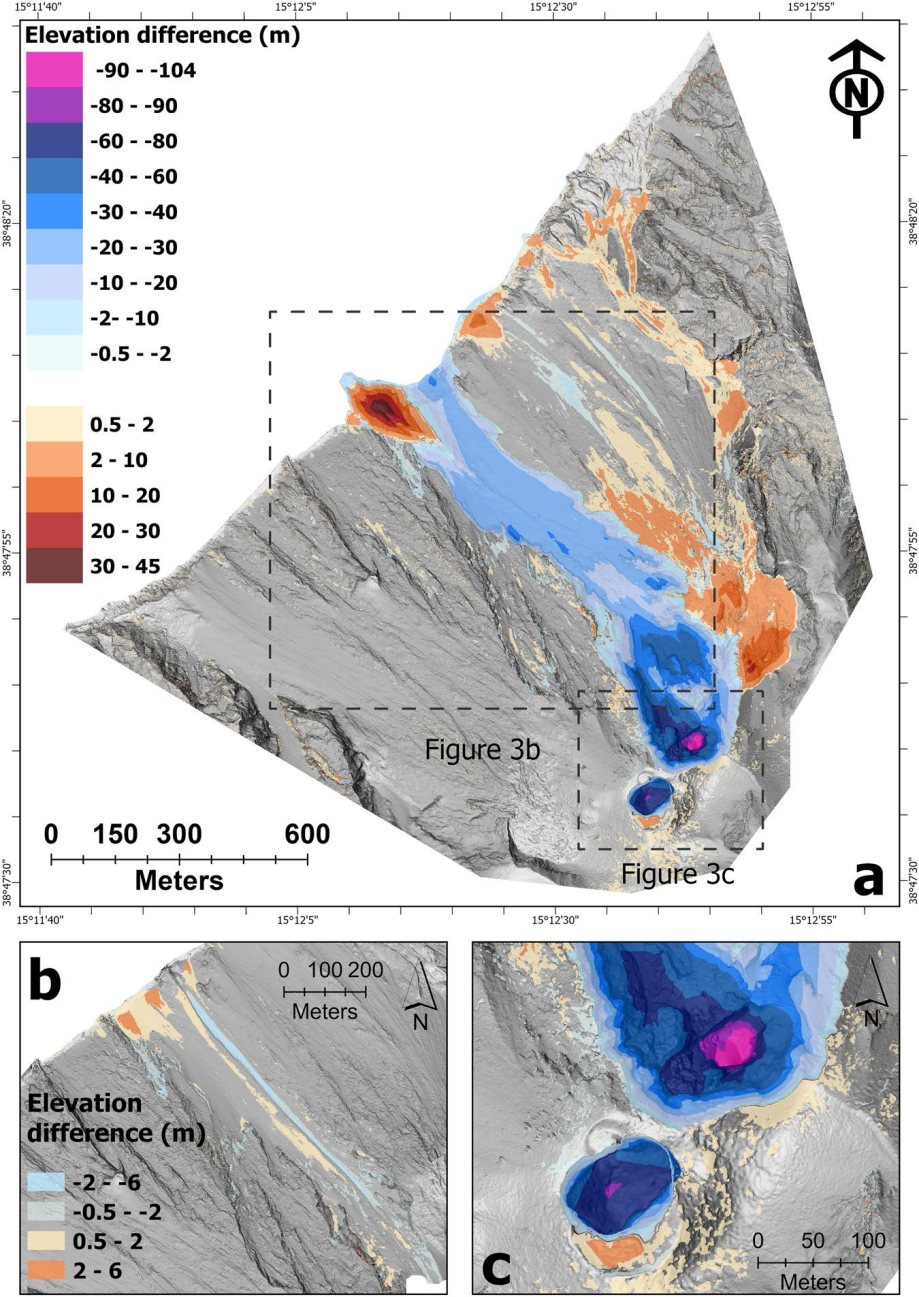
(**a**) Elevation difference map for the period 24 May 2024 - 14 July 2024 (pre- and post-July 2024 eruption). (**b**) Elevation difference map for the period 24 May 2024 - 04 July 2024^19^ (pre-eruption), showing no significant morphological changes in the period immediately preceding the 4–11 July 2024 eruption. (**c**) Detail of the elevation difference map (same symbology) presented in a).

We obtained elevation change detection and estimated volume change at Stromboli following the July 2024 eruption by differencing our 14 July 2024 survey^17^ (Fig. 2a) and a pre-event DSM resulting from a survey performed on 24 May 2024^18^. We used the freely available Geomorphic Change Detection (GCD) plugin for ArcGIS (see gcd.riverscapes.net/, accessed on 11 August 2024) to create the DEM of Difference (DoD) and set the threshold elevation change (minimum level of detection or minimum elevation change that can confidently be considered a true change) to 0.5 m. The DoD was calculated for the entire extent of Fig. 3a. The 24 May DSM is the most recent and complete survey that matches the area of our 14 July survey and can be confidently used as the reference dataset since no significant morphological changes have been observed in the period immediately preceding the 4–11 July 2024 eruption (Fig. 3b). Differencing the 14 July and 24 May DSMs produced a map of the elevation change within the study area (Fig. 3a), highlighting the profound morphological changes of the crater terrace and of the Sciara del Fuoco.

## Data Records

The data record consists of a high-resolution (0.2 m/pixel) photogrammetric Digital Surface Model processed from survey campaign photographs using Agisoft Metashape Pro®. Details of the photogrammetric survey data and elaboration are summarised in Table 1. The dataset is stored in GeoTIFF file format in the OpenTopography repository^17^ and is shared under the CC BY 4.0 use licence. Along with the GeoTIFF DSM, a point cloud in the .laz file format is available within the same repository.

## Technical Validation

We performed the validation of the data partially following Civico *et al*.^4^. Errors in our photogrammetrically-generated DSM result from a complex interplay of geometric and physical parameters, such as image scale, ground sampling density (GSD), camera network geometry (nadiral, cross, oblique strips), percentages of image overlap (forward and sidelap), camera shutter speed and exposure settings, lens specifications, image sharpness, camera calibrations, flight design (e.g., flight-line geometry and altitude), surface texture and albedo, lighting conditions, disturbances from volcanic activity, as well as on processing: SfM, BBA (Bundle Block Adjustment), image matching, point cloud noise, and outlier removal algorithms. We therefore applied several strategies to mitigate errors, among which the most important were the following: (1) the use of fast (>1/400 s) camera shutter speeds (i.e., exposure times) whenever possible, (2) the variation of flight altitudes and camera orientation, (3) the application of best practices for processing in Agisoft Metashape, (e.g.^1^), and (4) the removal of sparse cloud points with large uncertainty via Metashape’s gradual selection tool.

Measuring ground control points to scale and georeference the SfM point cloud and the derived DSM within the crater terrace and Sciara del Fuoco areas was not possible due to the ongoing eruption. Therefore, the surveys were initially scaled and georeferenced using GNSS-RTK information embedded in the image metadata, with an estimated camera locations total error of 6.3 cm for the visible images survey and 3.3 cm for the thermal images survey.

We further improved the spatial accuracy of our georeferenced 3D point cloud using the Cloud Compare open-source software (www.danielgm.net/cc/, accessed on 11 August 2024). In Cloud Compare, we aligned the 14 July 2024 point cloud to a point cloud derived from a 2023 airborne lidar survey of the entire Stromboli island (referenced to the ITALGEO 2005 geoid) using 10 evenly distributed manually selected equivalent point pairs in unchanged areas of the volcano (Fig. 1). The root mean square (RMS) error for the point cloud alignment is 0.26 metres.

## Data Availability

No custom code was used to generate or process the data described in the manuscript.
